# Book Reviews

**Published:** 1872-10-01

**Authors:** 


### Book Reviews.

The Science and Practice of Medicine, by William Aitken, M.D. Third American, from the Sixth London Edition. With additions by Meredith Clymer, M.D. Philadelphia: Lindsay & Blakiston.

The new edition of this favorite standard work on the Practice of Medicine comes to us in an enlarged as well as thoroughly revised form. The new material added in the present edition is equivalent in bulk to a third volume added to the last edition, yet the size of the work has not been materially increased; a reduction in the size of the type enabling a larger amount of matter to be condensed into the same space.

As the author states in the preface, he has endeavored to embody an account of all the more recent advances in the Science and Practice of Medicine, which during the past fourteen years have been unusually numerous and important. Many chapters have been to a great extent rewritten and remodelled, and many new topics have been added.

The subjects composing Part I have been greatly expanded by "topics relative to pathology and morbid anatomy." Numerous additions have also been made where topics of importance had only been shortly noticed before; while the sections on the Prevention and Treatment of Diseases have been more fully considered and expressed. The work has been to a great extent, indeed, rewritten; and descriptions of many diseases altogether omitted in former editions, are now introduced.

In this new edition the author has endeavored to adhere closely to the new nomenclature of the College of Physicians, and which has been adopted by the government authorities of Great Britain.

A Treatise on Diseases of the Bones. By Thos. M. Markoe, M.D., etc. New York: D. Appleton & Co.

This is a volume of 413 pages, profusely illustrated with wood cuts, etc. The work contains the substance of the lectures delivered by the author during the past twelve years at the College of Physicians and Surgeons, New York. The table of contents comprises the following topics: Part I, Diseases of Bone; Hypertrophy and Atrophy; Inflammation of Bone; Suppuration in Bone; Chronic Sinuous Abscess of Bone; Diffuse Suppuration; Osteo Myelitis; Rickets; Mollities Ossium; Fragilities Osseum; Caries; Necrosis. Part II, Tumors of Bones; Cartilaginous, Osseous, Fibrous, and Fibroid Tumors; Myeloid and Pulsating Tumors of Bone; Tumors of the Jaws. Part III, Malignant Diseases of Bone; Scirrhous or Hard Cancer; Medullary or Soft Cancer; Epithelial, Melanoid, Colloid, and Ostoid Cancers; Treatment of Malignant Disease of Bone.

The well-known reputation of the author, and particularly the large amount of time and attention which he has devoted to the development of this special department, is an abundant guarantee of the great value and importance of this new treatise.

Transactions of the Minnesota State Medical Society, 1872. Minneapolis: Johnson & Smith, Publishers.

Transactions of the Indiana State Medical Society, Twenty-second Annual Session, 1872.

A pamphlet of 176 pages containing a variety of interesting and valuable reports.

